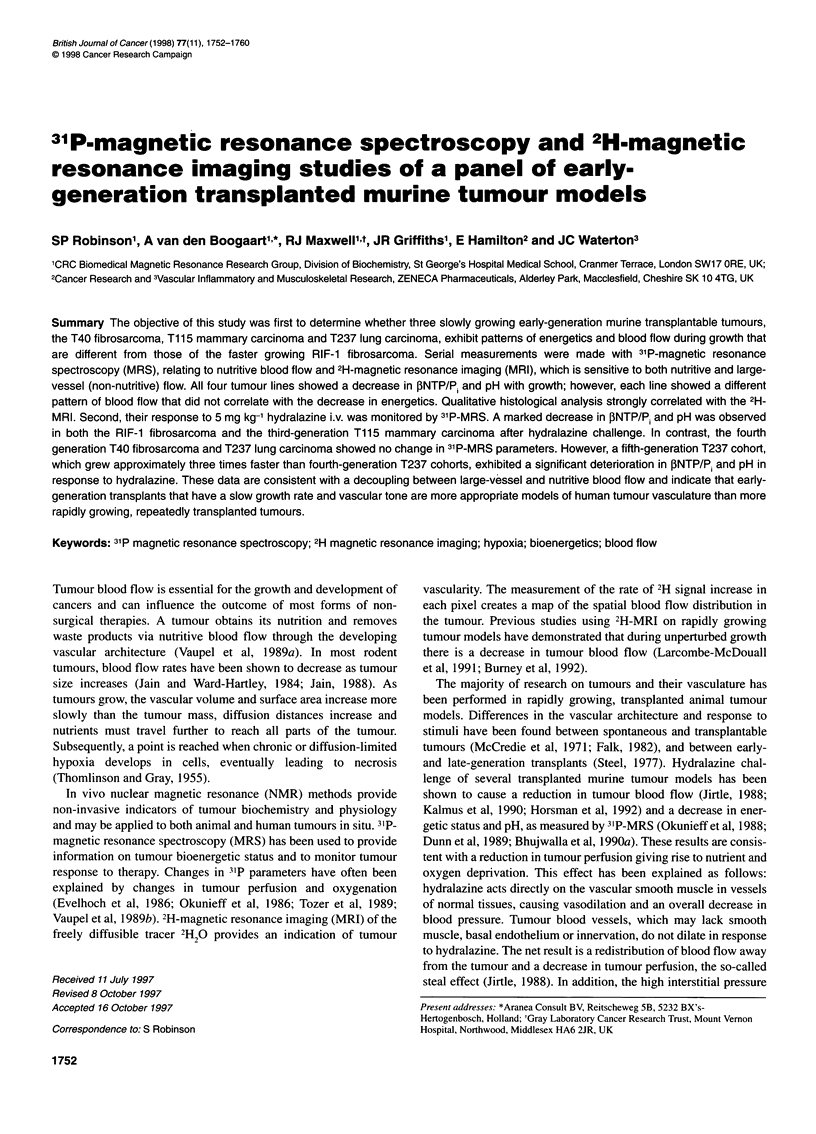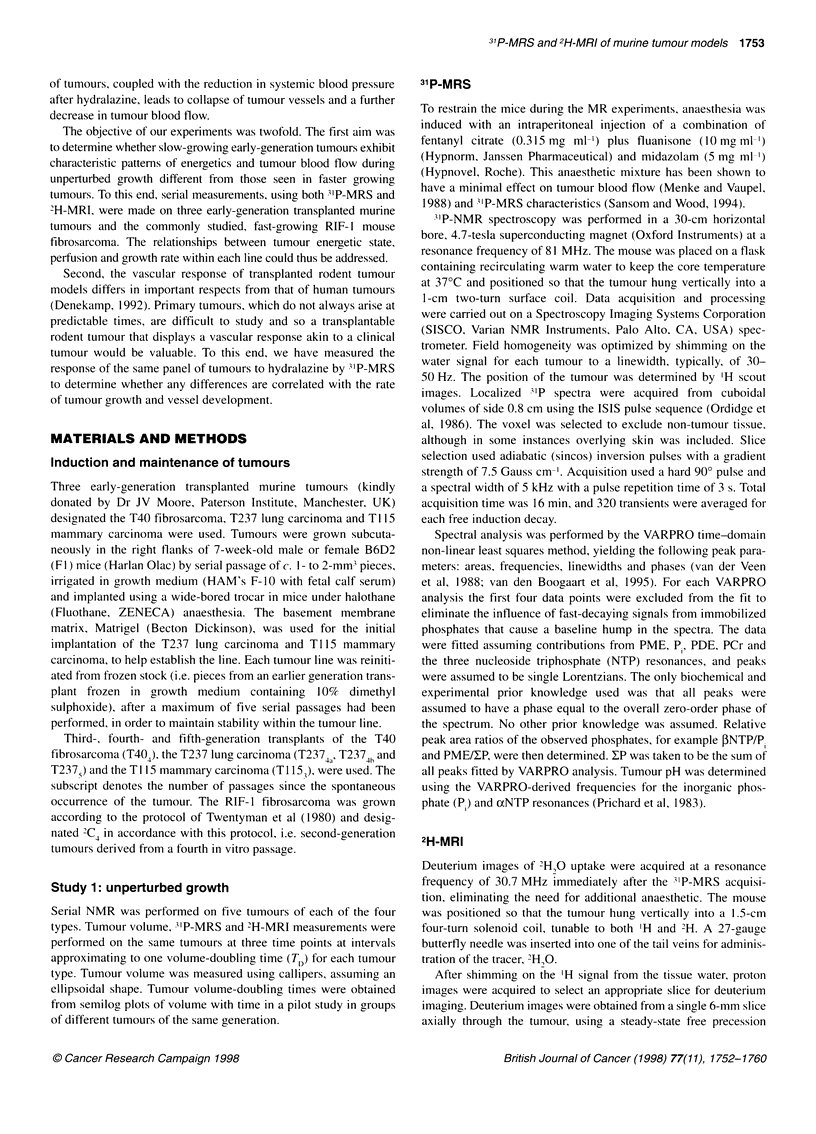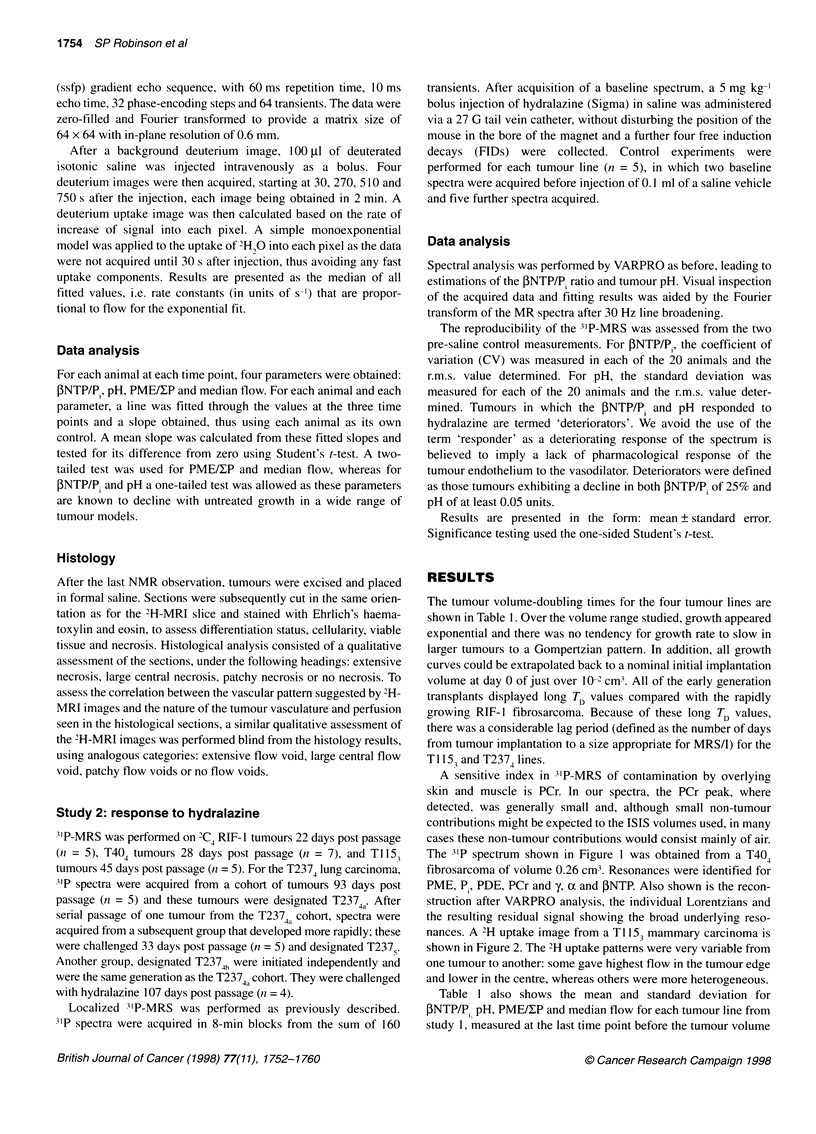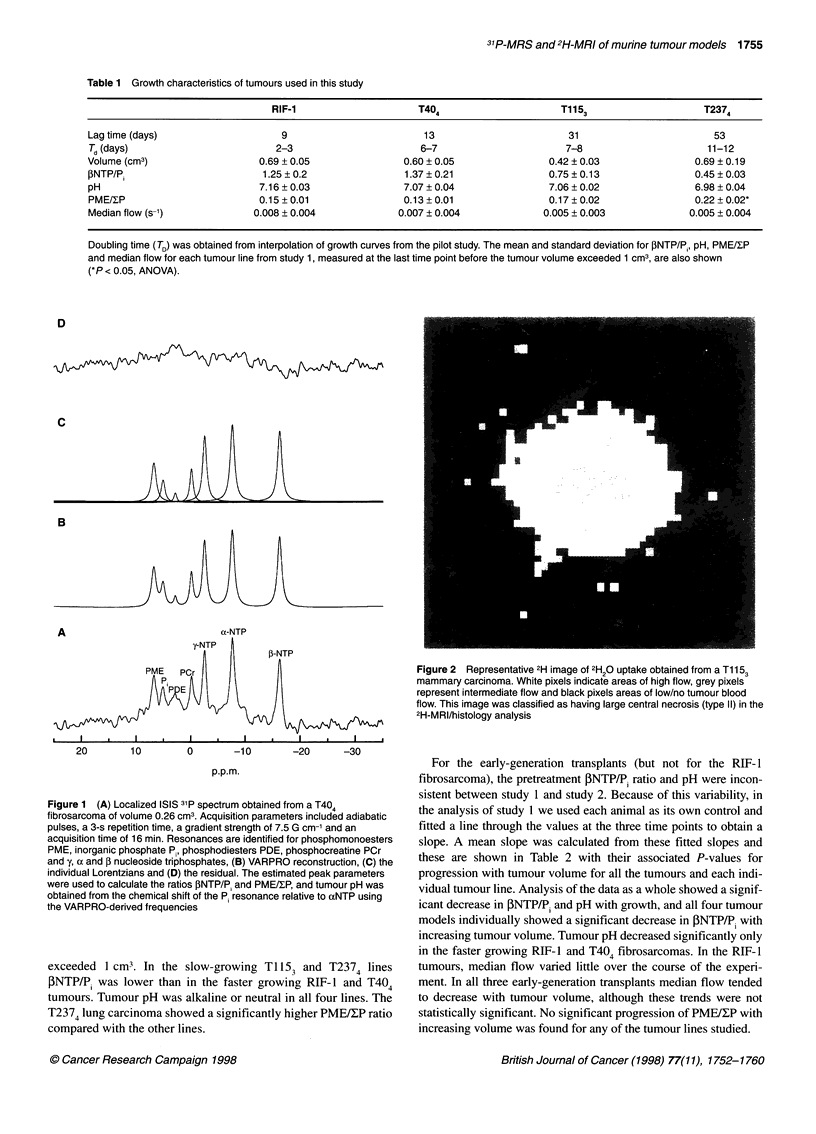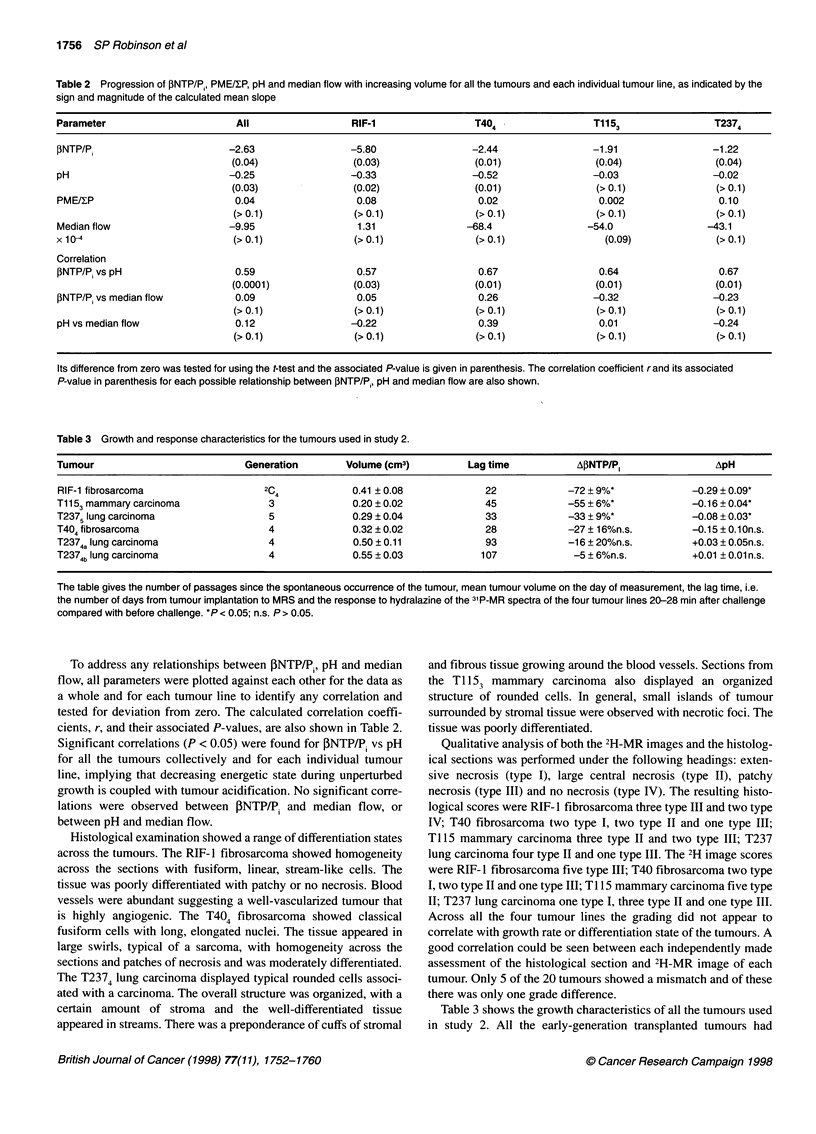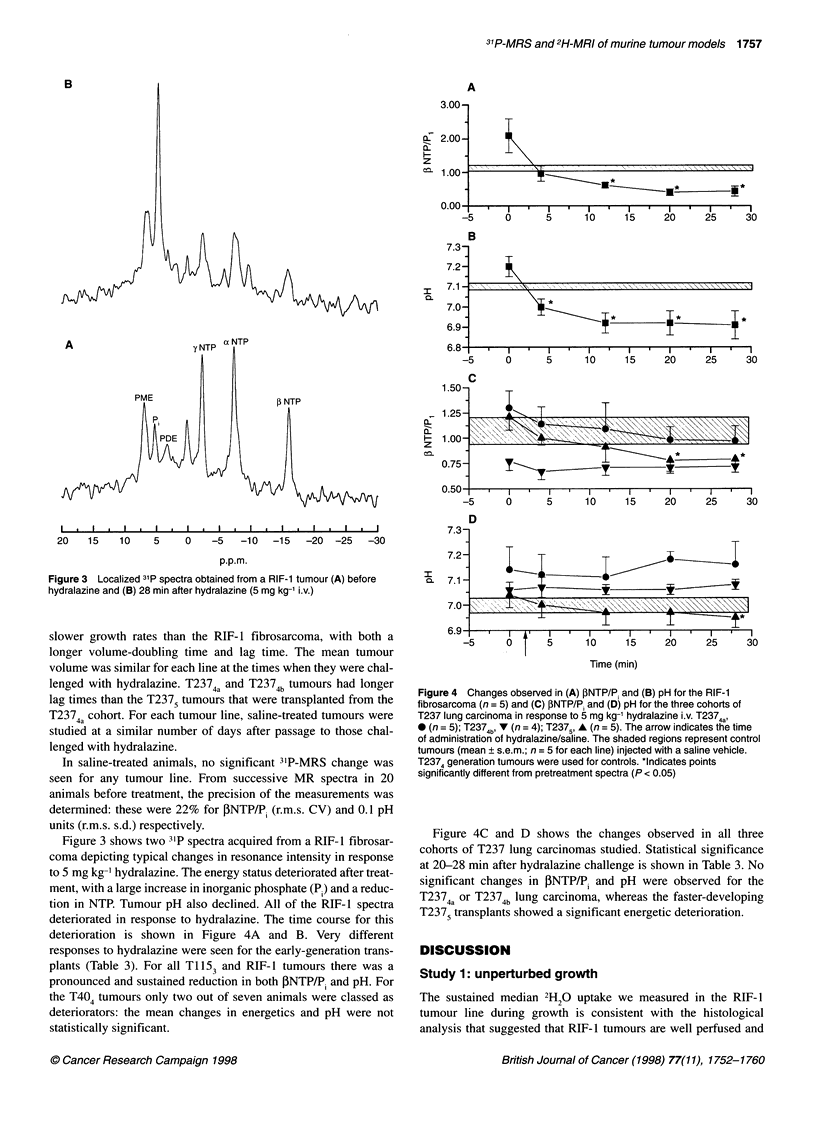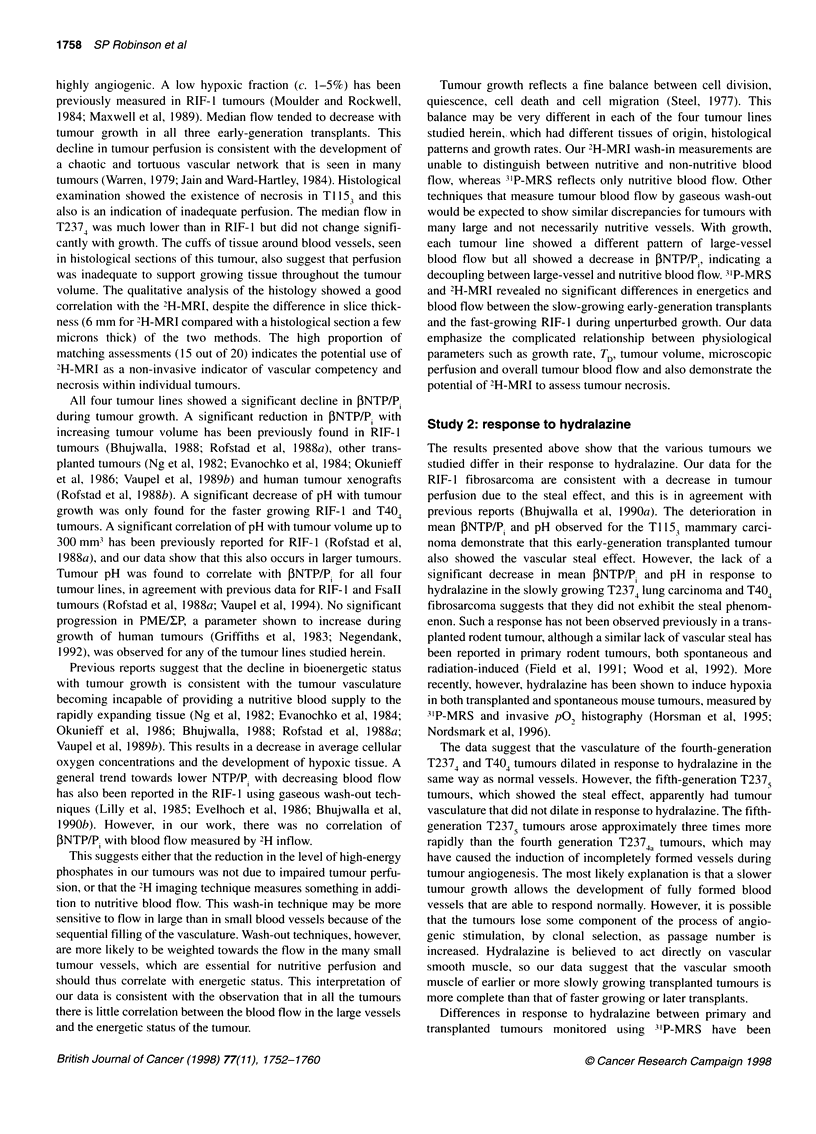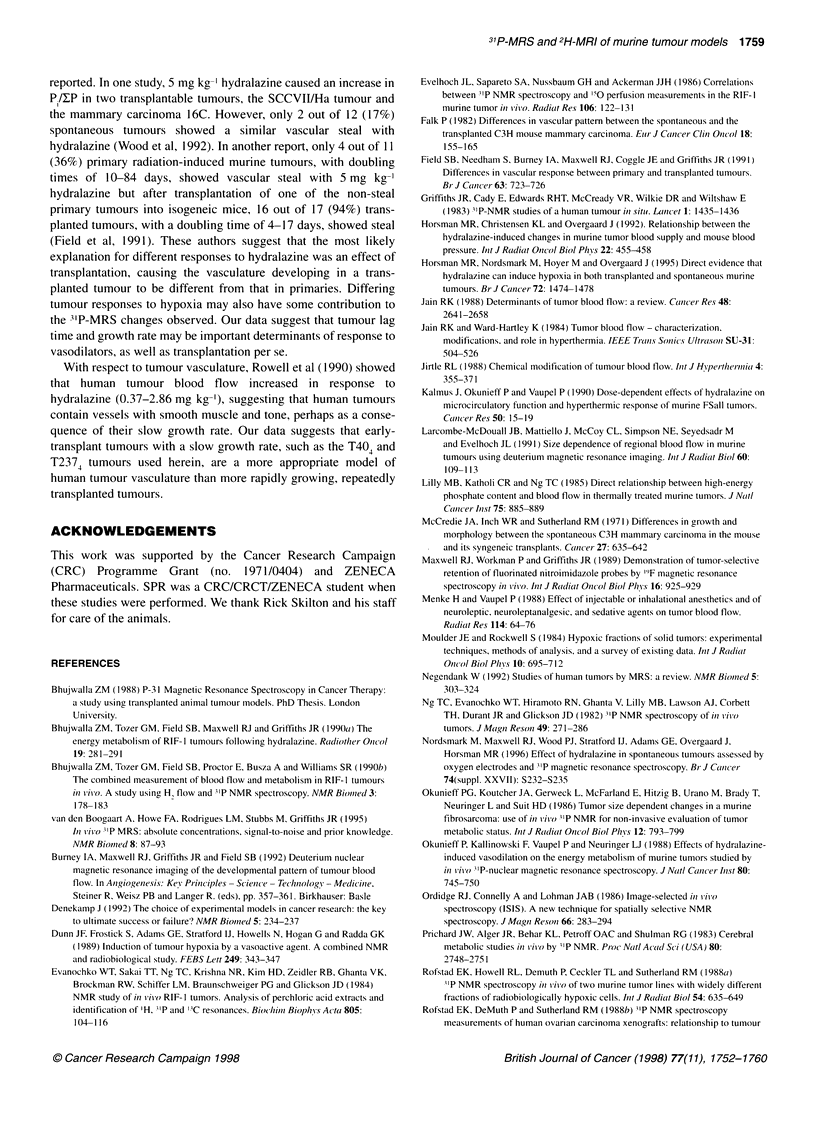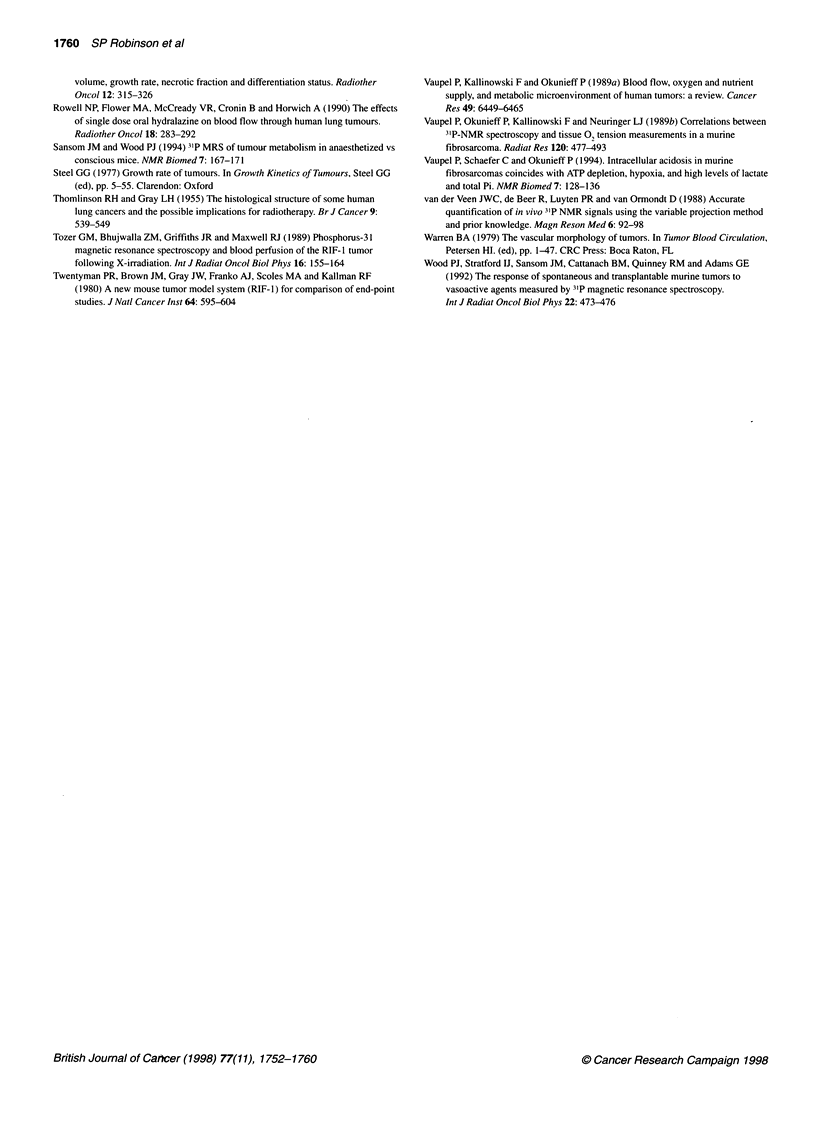# 31P-magnetic resonance spectroscopy and 2H-magnetic resonance imaging studies of a panel of early-generation transplanted murine tumour models.

**DOI:** 10.1038/bjc.1998.293

**Published:** 1998-06

**Authors:** S. P. Robinson, A. van den Boogaart, R. J. Maxwell, J. R. Griffiths, E. Hamilton, J. C. Waterton

**Affiliations:** CRC Biomedical Magnetic Resonance Research Group, Division of Biochemistry, St George's Hospital Medical School, London, UK.

## Abstract

**Images:**


British Journal of Cancer (1998) 77(11), 1752–1760
© 1998 Cancer Research Campaign

### $^{31}$P-magnetic resonance spectroscopy and $^{2}$H-magnetic resonance imaging studies of a panel of early-generation transplanted murine tumour models

**SP Robinson[1], A van den Boogaart[1,*], RJ Maxwell[1,†], JR Griffiths[1], E Hamilton[2] and JC Waterton[3]**

[1]CRC Biomedical Magnetic Resonance Research Group, Division of Biochemistry, St George's Hospital Medical School, Cranmer Terrace, London SW17 0RE, UK; [2]Cancer Research and [3]Vascular Inflammatory and Musculoskeletal Research, ZENECA Pharmaceuticals, Alderley Park, Macclesfield, Cheshire SK 10 4TG, UK

**Summary** The objective of this study was first to determine whether three slowly growing early-generation murine transplantable tumours, the T40 fibrosarcoma, T115 mammary carcinoma and T237 lung carcinoma, exhibit patterns of energetics and blood flow during growth that are different from those of the faster growing RIF-1 fibrosarcoma. Serial measurements were made with $^{31}$P-magnetic resonance spectroscopy (MRS), relating to nutritive blood flow and $^{2}$H-magnetic resonance imaging (MRI), which is sensitive to both nutritive and large-vessel (non-nutritive) flow. All four tumour lines showed a decrease in $\beta$NTP/$P_i$ and pH with growth; however, each line showed a different pattern of blood flow that did not correlate with the decrease in energetics. Qualitative histological analysis strongly correlated with the $^{2}$H-MRI. Second, their response to 5 mg kg$^{-1}$ hydralazine i.v. was monitored by $^{31}$P-MRS. A marked decrease in $\beta$NTP/$P_i$ and pH was observed in both the RIF-1 fibrosarcoma and the third-generation T115 mammary carcinoma after hydralazine challenge. In contrast, the fourth generation T40 fibrosarcoma and T237 lung carcinoma showed no change in $^{31}$P-MRS parameters. However, a fifth-generation T237 cohort, which grew approximately three times faster than fourth-generation T237 cohorts, exhibited a significant deterioration in $\beta$NTP/$P_i$ and pH in response to hydralazine. These data are consistent with a decoupling between large-vessel and nutritive blood flow and indicate that early-generation transplants that have a slow growth rate and vascular tone are more appropriate models of human tumour vasculature than more rapidly growing, repeatedly transplanted tumours.

**Keywords:** $^{31}$P magnetic resonance spectroscopy; $^{2}$H magnetic resonance imaging; hypoxia; bioenergetics; blood flow

Tumour blood flow is essential for the growth and development of cancers and can influence the outcome of most forms of non-surgical therapies. A tumour obtains its nutrition and removes waste products via nutritive blood flow through the developing vascular architecture (Vaupel et al, 1989*a*). In most rodent tumours, blood flow rates have been shown to decrease as tumour size increases (Jain and Ward-Hartley, 1984; Jain, 1988). As tumours grow, the vascular volume and surface area increase more slowly than the tumour mass, diffusion distances increase and nutrients must travel further to reach all parts of the tumour. Subsequently, a point is reached when chronic or diffusion-limited hypoxia develops in cells, eventually leading to necrosis (Thomlinson and Gray, 1955).

In vivo nuclear magnetic resonance (NMR) methods provide non-invasive indicators of tumour biochemistry and physiology and may be applied to both animal and human tumours in situ. $^{31}$P-magnetic resonance spectroscopy (MRS) has been used to provide information on tumour bioenergetic status and to monitor tumour response to therapy. Changes in $^{31}$P parameters have often been explained by changes in tumour perfusion and oxygenation (Evelhoch et al, 1986; Okunieff et al, 1986; Tozer et al, 1989; Vaupel et al, 1989*b*). $^{2}$H-magnetic resonance imaging (MRI) of the freely diffusible tracer $^{2}H_2O$ provides an indication of tumour vascularity. The measurement of the rate of $^{2}$H signal increase in each pixel creates a map of the spatial blood flow distribution in the tumour. Previous studies using $^{2}$H-MRI on rapidly growing tumour models have demonstrated that during unperturbed growth there is a decrease in tumour blood flow (Larcombe-McDouall et al, 1991; Burney et al, 1992).

The majority of research on tumours and their vasculature has been performed in rapidly growing, transplanted animal tumour models. Differences in the vascular architecture and response to stimuli have been found between spontaneous and transplantable tumours (McCredie et al, 1971; Falk, 1982), and between early- and late-generation transplants (Steel, 1977). Hydralazine challenge of several transplanted murine tumour models has been shown to cause a reduction in tumour blood flow (Jirtle, 1988; Kalmus et al, 1990; Horsman et al, 1992) and a decrease in energetic status and pH, as measured by $^{31}$P-MRS (Okunieff et al, 1988; Dunn et al, 1989; Bhujwalla et al, 1990*a*). These results are consistent with a reduction in tumour perfusion giving rise to nutrient and oxygen deprivation. This effect has been explained as follows: hydralazine acts directly on the vascular smooth muscle in vessels of normal tissues, causing vasodilation and an overall decrease in blood pressure. Tumour blood vessels, which may lack smooth muscle, basal endothelium or innervation, do not dilate in response to hydralazine. The net result is a redistribution of blood flow away from the tumour and a decrease in tumour perfusion, the so-called steal effect (Jirtle, 1988). In addition, the high interstitial pressure

*Received 11 July 1997*
*Revised 8 October 1997*
*Accepted 16 October 1997*

*Correspondence to:* S Robinson

*Present addresses:* *Aranea Consult BV, Reitscheweg 5B, 5232 BX's-Hertogenbosch, Holland; †Gray Laboratory Cancer Research Trust, Mount Vernon Hospital, Northwood, Middlesex HA6 2JR, UK

of tumours, coupled with the reduction in systemic blood pressure after hydralazine, leads to collapse of tumour vessels and a further decrease in tumour blood flow.

The objective of our experiments was twofold. The first aim was to determine whether slow-growing early-generation tumours exhibit characteristic patterns of energetics and tumour blood flow during unperturbed growth different from those seen in faster growing tumours. To this end, serial measurements, using both $^{31}$P-MRS and $^{2}$H-MRI, were made on three early-generation transplanted murine tumours and the commonly studied, fast-growing RIF-1 mouse fibrosarcoma. The relationships between tumour energetic state, perfusion and growth rate within each line could thus be addressed.

Second, the vascular response of transplanted rodent tumour models differs in important respects from that of human tumours (Denekamp, 1992). Primary tumours, which do not always arise at predictable times, are difficult to study and so a transplantable rodent tumour that displays a vascular response akin to a clinical tumour would be valuable. To this end, we have measured the response of the same panel of tumours to hydralazine by $^{31}$P-MRS to determine whether any differences are correlated with the rate of tumour growth and vessel development.

### MATERIALS AND METHODS

### Induction and maintenance of tumours

Three early-generation transplanted murine tumours (kindly donated by Dr JV Moore, Paterson Institute, Manchester, UK) designated the T40 fibrosarcoma, T237 lung carcinoma and T115 mammary carcinoma were used. Tumours were grown subcutaneously in the right flanks of 7-week-old male or female B6D2 (F1) mice (Harlan Olac) by serial passage of *c*. 1- to 2-mm$^3$ pieces, irrigated in growth medium (HAM's F-10 with fetal calf serum) and implanted using a wide-bored trocar in mice under halothane (Fluothane, ZENECA) anaesthesia. The basement membrane matrix, Matrigel (Becton Dickinson), was used for the initial implantation of the T237 lung carcinoma and T115 mammary carcinoma, to help establish the line. Each tumour line was reinitiated from frozen stock (i.e. pieces from an earlier generation transplant frozen in growth medium containing 10% dimethyl sulphoxide), after a maximum of five serial passages had been performed, in order to maintain stability within the tumour line.

Third-, fourth- and fifth-generation transplants of the T40 fibrosarcoma ($T40_4$), the T237 lung carcinoma ($T237_{4a}$, $T237_{4b}$ and $T237_5$) and the T115 mammary carcinoma ($T115_3$), were used. The subscript denotes the number of passages since the spontaneous occurrence of the tumour. The RIF-1 fibrosarcoma was grown according to the protocol of Twentyman et al (1980) and designated $^{2}C_4$ in accordance with this protocol, i.e. second-generation tumours derived from a fourth in vitro passage.

### Study 1: unperturbed growth

Serial NMR was performed on five tumours of each of the four types. Tumour volume, $^{31}$P-MRS and $^{2}$H-MRI measurements were performed on the same tumours at three time points at intervals approximating to one volume-doubling time ($T_D$) for each tumour type. Tumour volume was measured using callipers, assuming an ellipsoidal shape. Tumour volume-doubling times were obtained from semilog plots of volume with time in a pilot study in groups of different tumours of the same generation.

### $^{31}$P-MRS

To restrain the mice during the MR experiments, anaesthesia was induced with an intraperitoneal injection of a combination of fentanyl citrate (0.315 mg ml$^{-1}$) plus fluanisone (10 mg ml$^{-1}$) (Hypnorm, Janssen Pharmaceutical) and midazolam (5 mg ml$^{-1}$) (Hypnovel, Roche). This anaesthetic mixture has been shown to have a minimal effect on tumour blood flow (Menke and Vaupel, 1988) and $^{31}$P-MRS characteristics (Sansom and Wood, 1994).

$^{31}$P-NMR spectroscopy was performed in a 30-cm horizontal bore, 4.7-tesla superconducting magnet (Oxford Instruments) at a resonance frequency of 81 MHz. The mouse was placed on a flask containing recirculating warm water to keep the core temperature at 37°C and positioned so that the tumour hung vertically into a 1-cm two-turn surface coil. Data acquisition and processing were carried out on a Spectroscopy Imaging Systems Corporation (SISCO, Varian NMR Instruments, Palo Alto, CA, USA) spectrometer. Field homogeneity was optimized by shimming on the water signal for each tumour to a linewidth, typically, of 30–50 Hz. The position of the tumour was determined by $^{1}$H scout images. Localized $^{31}$P spectra were acquired from cuboidal volumes of side 0.8 cm using the ISIS pulse sequence (Ordidge et al, 1986). The voxel was selected to exclude non-tumour tissue, although in some instances overlying skin was included. Slice selection used adiabatic (sincos) inversion pulses with a gradient strength of 7.5 Gauss cm$^{-1}$. Acquisition used a hard 90° pulse and a spectral width of 5 kHz with a pulse repetition time of 3 s. Total acquisition time was 16 min, and 320 transients were averaged for each free induction decay.

Spectral analysis was performed by the VARPRO time–domain non-linear least squares method, yielding the following peak parameters: areas, frequencies, linewidths and phases (van der Veen et al, 1988; van den Boogaart et al, 1995). For each VARPRO analysis the first four data points were excluded from the fit to eliminate the influence of fast-decaying signals from immobilized phosphates that cause a baseline hump in the spectra. The data were fitted assuming contributions from PME, $P_i$, PDE, PCr and the three nucleoside triphosphate (NTP) resonances, and peaks were assumed to be single Lorentzians. The only biochemical and experimental prior knowledge used was that all peaks were assumed to have a phase equal to the overall zero-order phase of the spectrum. No other prior knowledge was assumed. Relative peak area ratios of the observed phosphates, for example $\beta NTP/P_i$ and $PME/\Sigma P$, were then determined. $\Sigma P$ was taken to be the sum of all peaks fitted by VARPRO analysis. Tumour pH was determined using the VARPRO-derived frequencies for the inorganic phosphate ($P_i$) and $\alpha$NTP resonances (Prichard et al, 1983).

### $^{2}$H-MRI

Deuterium images of $^{2}H_2O$ uptake were acquired at a resonance frequency of 30.7 MHz immediately after the $^{31}$P-MRS acquisition, eliminating the need for additional anaesthetic. The mouse was positioned so that the tumour hung vertically into a 1.5-cm four-turn solenoid coil, tunable to both $^{1}$H and $^{2}$H. A 27-gauge butterfly needle was inserted into one of the tail veins for administration of the tracer, $^{2}H_2O$.

After shimming on the $^{1}$H signal from the tissue water, proton images were acquired to select an appropriate slice for deuterium imaging. Deuterium images were obtained from a single 6-mm slice axially through the tumour, using a steady-state free precession

© Cancer Research Campaign 1998

(ssfp) gradient echo sequence, with 60 ms repetition time, 10 ms echo time, 32 phase-encoding steps and 64 transients. The data were zero-filled and Fourier transformed to provide a matrix size of 64 × 64 with in-plane resolution of 0.6 mm.

After a background deuterium image, 100 µl of deuterated isotonic saline was injected intravenously as a bolus. Four deuterium images were then acquired, starting at 30, 270, 510 and 750 s after the injection, each image being obtained in 2 min. A deuterium uptake image was then calculated based on the rate of increase of signal into each pixel. A simple monoexponential model was applied to the uptake of $^2H_2O$ into each pixel as the data were not acquired until 30 s after injection, thus avoiding any fast uptake components. Results are presented as the median of all fitted values, i.e. rate constants (in units of $s^{-1}$) that are proportional to flow for the exponential fit.

### Data analysis

For each animal at each time point, four parameters were obtained: $\beta$NTP/$P_i$, pH, PME/$\Sigma$P and median flow. For each animal and each parameter, a line was fitted through the values at the three time points and a slope obtained, thus using each animal as its own control. A mean slope was calculated from these fitted slopes and tested for its difference from zero using Student's *t*-test. A two-tailed test was used for PME/$\Sigma$P and median flow, whereas for $\beta$NTP/$P_i$ and pH a one-tailed test was allowed as these parameters are known to decline with untreated growth in a wide range of tumour models.

### Histology

After the last NMR observation, tumours were excised and placed in formal saline. Sections were subsequently cut in the same orientation as for the $^2$H-MRI slice and stained with Ehrlich's haematoxylin and eosin, to assess differentiation status, cellularity, viable tissue and necrosis. Histological analysis consisted of a qualitative assessment of the sections, under the following headings: extensive necrosis, large central necrosis, patchy necrosis or no necrosis. To assess the correlation between the vascular pattern suggested by $^2$H-MRI images and the nature of the tumour vasculature and perfusion seen in the histological sections, a similar qualitative assessment of the $^2$H-MRI images was performed blind from the histology results, using analogous categories: extensive flow void, large central flow void, patchy flow voids or no flow voids.

### Study 2: response to hydralazine

$^{31}$P-MRS was performed on $^2C_4$ RIF-1 tumours 22 days post passage ($n$ = 5), $T40_4$ tumours 28 days post passage ($n$ = 7), and $T115_3$ tumours 45 days post passage ($n$ = 5). For the $T237_4$ lung carcinoma, $^{31}$P spectra were acquired from a cohort of tumours 93 days post passage ($n$ = 5) and these tumours were designated $T237_{4a}$. After serial passage of one tumour from the $T237_{4a}$ cohort, spectra were acquired from a subsequent group that developed more rapidly; these were challenged 33 days post passage ($n$ = 5) and designated $T237_5$. Another group, designated $T237_{4b}$ were initiated independently and were the same generation as the $T237_{4a}$ cohort. They were challenged with hydralazine 107 days post passage ($n$ = 4).

Localized $^{31}$P-MRS was performed as previously described. $^{31}$P spectra were acquired in 8-min blocks from the sum of 160 transients. After acquisition of a baseline spectrum, a 5 mg $kg^{-1}$ bolus injection of hydralazine (Sigma) in saline was administered via a 27 G tail vein catheter, without disturbing the position of the mouse in the bore of the magnet and a further four free induction decays (FIDs) were collected. Control experiments were performed for each tumour line ($n$ = 5), in which two baseline spectra were acquired before injection of 0.1 ml of a saline vehicle and five further spectra acquired.

### Data analysis

Spectral analysis was performed by VARPRO as before, leading to estimations of the $\beta$NTP/$P_i$ ratio and tumour pH. Visual inspection of the acquired data and fitting results was aided by the Fourier transform of the MR spectra after 30 Hz line broadening.

The reproducibility of the $^{31}$P-MRS was assessed from the two pre-saline control measurements. For $\beta$NTP/$P_i$, the coefficient of variation (CV) was measured in each of the 20 animals and the r.m.s. value determined. For pH, the standard deviation was measured for each of the 20 animals and the r.m.s. value determined. Tumours in which the $\beta$NTP/$P_i$ and pH responded to hydralazine are termed 'deteriorators'. We avoid the use of the term 'responder' as a deteriorating response of the spectrum is believed to imply a lack of pharmacological response of the tumour endothelium to the vasodilator. Deteriorators were defined as those tumours exhibiting a decline in both $\beta$NTP/$P_i$ of 25% and pH of at least 0.05 units.

Results are presented in the form: mean ± standard error. Significance testing used the one-sided Student's *t*-test.

### RESULTS

The tumour volume-doubling times for the four tumour lines are shown in Table 1. Over the volume range studied, growth appeared exponential and there was no tendency for growth rate to slow in larger tumours to a Gompertzian pattern. In addition, all growth curves could be extrapolated back to a nominal initial implantation volume at day 0 of just over $10^{-2}$ $cm^3$. All of the early generation transplants displayed long $T_D$ values compared with the rapidly growing RIF-1 fibrosarcoma. Because of these long $T_D$ values, there was a considerable lag period (defined as the number of days from tumour implantation to a size appropriate for MRS/I) for the $T115_3$ and $T237_4$ lines.

A sensitive index in $^{31}$P-MRS of contamination by overlying skin and muscle is PCr. In our spectra, the PCr peak, where detected, was generally small and, although small non-tumour contributions might be expected to the ISIS volumes used, in many cases these non-tumour contributions would consist mainly of air. The $^{31}$P spectrum shown in Figure 1 was obtained from a $T40_4$ fibrosarcoma of volume 0.26 $cm^3$. Resonances were identified for PME, $P_i$, PDE, PCr and $\gamma$, $\alpha$ and $\beta$NTP. Also shown is the reconstruction after VARPRO analysis, the individual Lorentzians and the resulting residual signal showing the broad underlying resonances. A $^2$H uptake image from a $T115_3$ mammary carcinoma is shown in Figure 2. The $^2$H uptake patterns were very variable from one tumour to another: some gave highest flow in the tumour edge and lower in the centre, whereas others were more heterogeneous.

Table 1 also shows the mean and standard deviation for $\beta$NTP/$P_i$, pH, PME/$\Sigma$P and median flow for each tumour line from study 1, measured at the last time point before the tumour volume

© Cancer Research Campaign 1998

**Table 1** Growth characteristics of tumours used in this study

| | RIF-1 | $T40_4$ | $T115_3$ | $T237_4$ |
|---|---|---|---|---|
| Lag time (days) | 9 | 13 | 31 | 53 |
| $T_d$ (days) | 2–3 | 6–7 | 7–8 | 11–12 |
| Volume (cm³) | 0.69 ± 0.05 | 0.60 ± 0.05 | 0.42 ± 0.03 | 0.69 ± 0.19 |
| βNTP/$P_i$ | 1.25 ± 0.2 | 1.37 ± 0.21 | 0.75 ± 0.13 | 0.45 ± 0.03 |
| pH | 7.16 ± 0.03 | 7.07 ± 0.04 | 7.06 ± 0.02 | 6.98 ± 0.04 |
| PME/ΣP | 0.15 ± 0.01 | 0.13 ± 0.01 | 0.17 ± 0.02 | 0.22 ± 0.02* |
| Median flow (s⁻¹) | 0.008 ± 0.004 | 0.007 ± 0.004 | 0.005 ± 0.003 | 0.005 ± 0.004 |

Doubling time ($T_D$) was obtained from interpolation of growth curves from the pilot study. The mean and standard deviation for βNTP/$P_i$, pH, PME/ΣP and median flow for each tumour line from study 1, measured at the last time point before the tumour volume exceeded 1 cm³, are also shown (*$P < 0.05$, ANOVA).

**Figure 1** (**A**) Localized ISIS $^{31}$P spectrum obtained from a $T40_4$ fibrosarcoma of volume 0.26 cm³. Acquisition parameters included adiabatic pulses, a 3-s repetition time, a gradient strength of 7.5 G cm⁻¹ and an acquisition time of 16 min. Resonances are identified for phosphomonoesters PME, inorganic phosphate $P_i$, phosphodiesters PDE, phosphocreatine PCr and γ, α and β nucleoside triphosphates, (**B**) VARPRO reconstruction, (**C**) the individual Lorentzians and (**D**) the residual. The estimated peak parameters were used to calculate the ratios βNTP/$P_i$ and PME/ΣP, and tumour pH was obtained from the chemical shift of the $P_i$ resonance relative to αNTP using the VARPRO-derived frequencies

**Figure 2** Representative $^{2}$H image of $^{2}H_2O$ uptake obtained from a $T115_3$ mammary carcinoma. White pixels indicate areas of high flow, grey pixels represent intermediate flow and black pixels areas of low/no tumour blood flow. This image was classified as having large central necrosis (type II) in the $^{2}$H-MRI/histology analysis exceeded 1 cm³. In the slow-growing $T115_3$ and $T237_4$ lines βNTP/$P_i$ was lower than in the faster growing RIF-1 and $T40_4$ tumours. Tumour pH was alkaline or neutral in all four lines. The $T237_4$ lung carcinoma showed a significantly higher PME/ΣP ratio compared with the other lines.

For the early-generation transplants (but not for the RIF-1 fibrosarcoma), the pretreatment βNTP/$P_i$ ratio and pH were inconsistent between study 1 and study 2. Because of this variability, in the analysis of study 1 we used each animal as its own control and fitted a line through the values at the three time points to obtain a slope. A mean slope was calculated from these fitted slopes and these are shown in Table 2 with their associated *P*-values for progression with tumour volume for all the tumours and each individual tumour line. Analysis of the data as a whole showed a significant decrease in βNTP/$P_i$ and pH with growth, and all four tumour models individually showed a significant decrease in βNTP/$P_i$ with increasing tumour volume. Tumour pH decreased significantly only in the faster growing RIF-1 and $T40_4$ fibrosarcomas. In the RIF-1 tumours, median flow varied little over the course of the experiment. In all three early-generation transplants median flow tended to decrease with tumour volume, although these trends were not statistically significant. No significant progression of PME/ΣP with increasing volume was found for any of the tumour lines studied.

**Table 2** Progression of $\beta$NTP/$P_i$, PME/$\Sigma$P, pH and median flow with increasing volume for all the tumours and each individual tumour line, as indicated by the sign and magnitude of the calculated mean slope

| Parameter | All | RIF-1 | $T40_4$ | $T115_3$ | $T237_4$ |
|---|---|---|---|---|---|
| $\beta$NTP/$P_i$ | −2.63 | −5.80 | −2.44 | −1.91 | −1.22 |
| | (0.04) | (0.03) | (0.01) | (0.04) | (0.04) |
| pH | −0.25 | −0.33 | −0.52 | −0.03 | −0.02 |
| | (0.03) | (0.02) | (0.01) | (> 0.1) | (> 0.1) |
| PME/$\Sigma$P | 0.04 | 0.08 | 0.02 | 0.002 | 0.10 |
| | (> 0.1) | (> 0.1) | (> 0.1) | (> 0.1) | (> 0.1) |
| Median flow | −9.95 | 1.31 | −68.4 | −54.0 | −43.1 |
| $\times 10^{-4}$ | (> 0.1) | (> 0.1) | (> 0.1) | (0.09) | (> 0.1) |
| Correlation | | | | | |
| $\beta$NTP/$P_i$ vs pH | 0.59 | 0.57 | 0.67 | 0.64 | 0.67 |
| | (0.0001) | (0.03) | (0.01) | (0.01) | (0.01) |
| $\beta$NTP/$P_i$ vs median flow | 0.09 | 0.05 | 0.26 | −0.32 | −0.23 |
| | (> 0.1) | (> 0.1) | (> 0.1) | (> 0.1) | (> 0.1) |
| pH vs median flow | 0.12 | −0.22 | 0.39 | 0.01 | −0.24 |
| | (> 0.1) | (> 0.1) | (> 0.1) | (> 0.1) | (> 0.1) |

Its difference from zero was tested for using the *t*-test and the associated *P*-value is given in parenthesis. The correlation coefficient *r* and its associated *P*-value in parenthesis for each possible relationship between $\beta$NTP/$P_i$, pH and median flow are also shown.

**Table 3** Growth and response characteristics for the tumours used in study 2.

| Tumour | Generation | Volume (cm³) | Lag time | $\Delta\beta$NTP/$P_i$ | $\Delta$pH |
|---|---|---|---|---|---|
| RIF-1 fibrosarcoma | $^{2}C_4$ | 0.41 ± 0.08 | 22 | −72 ± 9%* | −0.29 ± 0.09* |
| $T115_3$ mammary carcinoma | 3 | 0.20 ± 0.02 | 45 | −55 ± 6%* | −0.16 ± 0.04* |
| $T237_5$ lung carcinoma | 5 | 0.29 ± 0.04 | 33 | −33 ± 9%* | −0.08 ± 0.03* |
| $T40_4$ fibrosarcoma | 4 | 0.32 ± 0.02 | 28 | −27 ± 16%n.s. | −0.15 ± 0.10n.s. |
| $T237_{4a}$ lung carcinoma | 4 | 0.50 ± 0.11 | 93 | −16 ± 20%n.s. | +0.03 ± 0.05n.s. |
| $T237_{4b}$ lung carcinoma | 4 | 0.55 ± 0.03 | 107 | −5 ± 6%n.s. | +0.01 ± 0.01n.s. |

The table gives the number of passages since the spontaneous occurrence of the tumour, mean tumour volume on the day of measurement, the lag time, i.e. the number of days from tumour implantation to MRS and the response to hydralazine of the $^{31}$P-MR spectra of the four tumour lines 20–28 min after challenge compared with before challenge. **P* < 0.05; n.s. *P* > 0.05.

To address any relationships between $\beta$NTP/$P_i$, pH and median flow, all parameters were plotted against each other for the data as a whole and for each tumour line to identify any correlation and tested for deviation from zero. The calculated correlation coefficients, *r*, and their associated *P*-values, are also shown in Table 2. Significant correlations ($P < 0.05$) were found for $\beta$NTP/$P_i$ vs pH for all the tumours collectively and for each individual tumour line, implying that decreasing energetic state during unperturbed growth is coupled with tumour acidification. No significant correlations were observed between $\beta$NTP/$P_i$ and median flow, or between pH and median flow.

Histological examination showed a range of differentiation states across the tumours. The RIF-1 fibrosarcoma showed homogeneity across the sections with fusiform, linear, stream-like cells. The tissue was poorly differentiated with patchy or no necrosis. Blood vessels were abundant suggesting a well-vascularized tumour that is highly angiogenic. The $T40_4$ fibrosarcoma showed classical fusiform cells with long, elongated nuclei. The tissue appeared in large swirls, typical of a sarcoma, with homogeneity across the sections and patches of necrosis and was moderately differentiated. The $T237_4$ lung carcinoma displayed typical rounded cells associated with a carcinoma. The overall structure was organized, with a certain amount of stroma and the well-differentiated tissue appeared in streams. There was a preponderance of cuffs of stromal and fibrous tissue growing around the blood vessels. Sections from the $T115_3$ mammary carcinoma also displayed an organized structure of rounded cells. In general, small islands of tumour surrounded by stromal tissue were observed with necrotic foci. The tissue was poorly differentiated.

Qualitative analysis of both the $^{2}$H-MR images and the histological sections was performed under the following headings: extensive necrosis (type I), large central necrosis (type II), patchy necrosis (type III) and no necrosis (type IV). The resulting histological scores were RIF-1 fibrosarcoma three type III and two type IV; T40 fibrosarcoma two type I, two type II and one type III; T115 mammary carcinoma three type II and two type III; T237 lung carcinoma four type II and one type III. The $^{2}$H image scores were RIF-1 fibrosarcoma five type III; T40 fibrosarcoma two type I, two type II and one type III; T115 mammary carcinoma five type II; T237 lung carcinoma one type I, three type II and one type III. Across all the four tumour lines the grading did not appear to correlate with growth rate or differentiation state of the tumours. A good correlation could be seen between each independently made assessment of the histological section and $^{2}$H-MR image of each tumour. Only 5 of the 20 tumours showed a mismatch and of these there was only one grade difference.

Table 3 shows the growth characteristics of all the tumours used in study 2. All the early-generation transplanted tumours had

© Cancer Research Campaign 1998

**Figure 3** Localized $^{31}$P spectra obtained from a RIF-1 tumour (**A**) before hydralazine and (**B**) 28 min after hydralazine (5 mg kg$^{-1}$ i.v.)

**Figure 4** Changes observed in (**A**) βNTP/$P_i$ and (**B**) pH for the RIF-1 fibrosarcoma ($n$ = 5) and (**C**) βNTP/$P_i$ and (**D**) pH for the three cohorts of T237 lung carcinoma in response to 5 mg kg$^{-1}$ hydralazine i.v. $T237_{4a}$, ● ($n$ = 5); $T237_{4b}$, ▼ ($n$ = 4); $T237_5$, ▲ ($n$ = 5). The arrow indicates the time of administration of hydralazine/saline. The shaded regions represent control tumours (mean ± s.e.m.; $n$ = 5 for each line) injected with a saline vehicle. $T237_4$ generation tumours were used for controls. *Indicates points significantly different from pretreatment spectra ($P$ < 0.05)

slower growth rates than the RIF-1 fibrosarcoma, with both a longer volume-doubling time and lag time. The mean tumour volume was similar for each line at the times when they were challenged with hydralazine. $T237_{4a}$ and $T237_{4b}$ tumours had longer lag times than the $T237_5$ tumours that were transplanted from the $T237_{4a}$ cohort. For each tumour line, saline-treated tumours were studied at a similar number of days after passage to those challenged with hydralazine.

In saline-treated animals, no significant $^{31}$P-MRS change was seen for any tumour line. From successive MR spectra in 20 animals before treatment, the precision of the measurements was determined: these were 22% for βNTP/$P_i$ (r.m.s. CV) and 0.1 pH units (r.m.s. s.d.) respectively.

Figure 3 shows two $^{31}$P spectra acquired from a RIF-1 fibrosarcoma depicting typical changes in resonance intensity in response to 5 mg kg$^{-1}$ hydralazine. The energy status deteriorated after treatment, with a large increase in inorganic phosphate ($P_i$) and a reduction in NTP. Tumour pH also declined. All of the RIF-1 spectra deteriorated in response to hydralazine. The time course for this deterioration is shown in Figure 4A and B. Very different responses to hydralazine were seen for the early-generation transplants (Table 3). For all $T115_3$ and RIF-1 tumours there was a pronounced and sustained reduction in both βNTP/$P_i$ and pH. For the $T40_4$ tumours only two out of seven animals were classed as deteriorators: the mean changes in energetics and pH were not statistically significant.

Figure 4C and D shows the changes observed in all three cohorts of T237 lung carcinomas studied. Statistical significance at 20–28 min after hydralazine challenge is shown in Table 3. No significant changes in βNTP/$P_i$ and pH were observed for the $T237_{4a}$ or $T237_{4b}$ lung carcinoma, whereas the faster-developing $T237_5$ transplants showed a significant energetic deterioration.

### DISCUSSION

### Study 1: unperturbed growth

The sustained median $^{2}H_2O$ uptake we measured in the RIF-1 tumour line during growth is consistent with the histological analysis that suggested that RIF-1 tumours are well perfused and

© Cancer Research Campaign 1998

highly angiogenic. A low hypoxic fraction (*c.* 1–5%) has been previously measured in RIF-1 tumours (Moulder and Rockwell, 1984; Maxwell et al, 1989). Median flow tended to decrease with tumour growth in all three early-generation transplants. This decline in tumour perfusion is consistent with the development of a chaotic and tortuous vascular network that is seen in many tumours (Warren, 1979; Jain and Ward-Hartley, 1984). Histological examination showed the existence of necrosis in $T115_3$ and this also is an indication of inadequate perfusion. The median flow in $T237_4$ was much lower than in RIF-1 but did not change significantly with growth. The cuffs of tissue around blood vessels, seen in histological sections of this tumour, also suggest that perfusion was inadequate to support growing tissue throughout the tumour volume. The qualitative analysis of the histology showed a good correlation with the $^2$H-MRI, despite the difference in slice thickness (6 mm for $^2$H-MRI compared with a histological section a few microns thick) of the two methods. The high proportion of matching assessments (15 out of 20) indicates the potential use of $^2$H-MRI as a non-invasive indicator of vascular competency and necrosis within individual tumours.

All four tumour lines showed a significant decline in $\beta NTP/P_i$ during tumour growth. A significant reduction in $\beta NTP/P_i$ with increasing tumour volume has been previously found in RIF-1 tumours (Bhujwalla, 1988; Rofstad et al, 1988*a*), other transplanted tumours (Ng et al, 1982; Evanochko et al, 1984; Okunieff et al, 1986; Vaupel et al, 1989*b*) and human tumour xenografts (Rofstad et al, 1988*b*). A significant decrease of pH with tumour growth was only found for the faster growing RIF-1 and $T40_4$ tumours. A significant correlation of pH with tumour volume up to 300 $mm^3$ has been previously reported for RIF-1 (Rofstad et al, 1988*a*), and our data show that this also occurs in larger tumours. Tumour pH was found to correlate with $\beta NTP/P_i$ for all four tumour lines, in agreement with previous data for RIF-1 and FsaII tumours (Rofstad et al, 1988*a*; Vaupel et al, 1994). No significant progression in PME/ΣP, a parameter shown to increase during growth of human tumours (Griffiths et al, 1983; Negendank, 1992), was observed for any of the tumour lines studied herein.

Previous reports suggest that the decline in bioenergetic status with tumour growth is consistent with the tumour vasculature becoming incapable of providing a nutritive blood supply to the rapidly expanding tissue (Ng et al, 1982; Evanochko et al, 1984; Okunieff et al, 1986; Bhujwalla, 1988; Rofstad et al, 1988*a*; Vaupel et al, 1989*b*). This results in a decrease in average cellular oxygen concentrations and the development of hypoxic tissue. A general trend towards lower $NTP/P_i$ with decreasing blood flow has also been reported in the RIF-1 using gaseous wash-out techniques (Lilly et al, 1985; Evelhoch et al, 1986; Bhujwalla et al, 1990*b*). However, in our work, there was no correlation of $\beta NTP/P_i$ with blood flow measured by $^2$H inflow.

This suggests either that the reduction in the level of high-energy phosphates in our tumours was not due to impaired tumour perfusion, or that the $^2$H imaging technique measures something in addition to nutritive blood flow. This wash-in technique may be more sensitive to flow in large than in small blood vessels because of the sequential filling of the vasculature. Wash-out techniques, however, are more likely to be weighted towards the flow in the many small tumour vessels, which are essential for nutritive perfusion and should thus correlate with energetic status. This interpretation of our data is consistent with the observation that in all the tumours there is little correlation between the blood flow in the large vessels and the energetic status of the tumour.

Tumour growth reflects a fine balance between cell division, quiescence, cell death and cell migration (Steel, 1977). This balance may be very different in each of the four tumour lines studied herein, which had different tissues of origin, histological patterns and growth rates. Our $^2$H-MRI wash-in measurements are unable to distinguish between nutritive and non-nutritive blood flow, whereas $^{31}$P-MRS reflects only nutritive blood flow. Other techniques that measure tumour blood flow by gaseous wash-out would be expected to show similar discrepancies for tumours with many large and not necessarily nutritive vessels. With growth, each tumour line showed a different pattern of large-vessel blood flow but all showed a decrease in $\beta NTP/P_i$, indicating a decoupling between large-vessel and nutritive blood flow. $^{31}$P-MRS and $^2$H-MRI revealed no significant differences in energetics and blood flow between the slow-growing early-generation transplants and the fast-growing RIF-1 during unperturbed growth. Our data emphasize the complicated relationship between physiological parameters such as growth rate, $T_D$, tumour volume, microscopic perfusion and overall tumour blood flow and also demonstrate the potential of $^2$H-MRI to assess tumour necrosis.

### Study 2: response to hydralazine

The results presented above show that the various tumours we studied differ in their response to hydralazine. Our data for the RIF-1 fibrosarcoma are consistent with a decrease in tumour perfusion due to the steal effect, and this is in agreement with previous reports (Bhujwalla et al, 1990*a*). The deterioration in mean $\beta NTP/P_i$ and pH observed for the $T115_3$ mammary carcinoma demonstrate that this early-generation transplanted tumour also showed the vascular steal effect. However, the lack of a significant decrease in mean $\beta NTP/P_i$ and pH in response to hydralazine in the slowly growing $T237_4$ lung carcinoma and $T40_4$ fibrosarcoma suggests that they did not exhibit the steal phenomenon. Such a response has not been observed previously in a transplanted rodent tumour, although a similar lack of vascular steal has been reported in primary rodent tumours, both spontaneous and radiation-induced (Field et al, 1991; Wood et al, 1992). More recently, however, hydralazine has been shown to induce hypoxia in both transplanted and spontaneous mouse tumours, measured by $^{31}$P-MRS and invasive $pO_2$ histography (Horsman et al, 1995; Nordsmark et al, 1996).

The data suggest that the vasculature of the fourth-generation $T237_4$ and $T40_4$ tumours dilated in response to hydralazine in the same way as normal vessels. However, the fifth-generation $T237_5$ tumours, which showed the steal effect, apparently had tumour vasculature that did not dilate in response to hydralazine. The fifth-generation $T237_5$ tumours arose approximately three times more rapidly than the fourth generation $T237_{4a}$ tumours, which may have caused the induction of incompletely formed vessels during tumour angiogenesis. The most likely explanation is that a slower tumour growth allows the development of fully formed blood vessels that are able to respond normally. However, it is possible that the tumours lose some component of the process of angiogenic stimulation, by clonal selection, as passage number is increased. Hydralazine is believed to act directly on vascular smooth muscle, so our data suggest that the vascular smooth muscle of earlier or more slowly growing transplanted tumours is more complete than that of faster growing or later transplants.

Differences in response to hydralazine between primary and transplanted tumours monitored using $^{31}$P-MRS have been

© Cancer Research Campaign 1998

reported. In one study, 5 mg kg$^{-1}$ hydralazine caused an increase in $P_i/\Sigma P$ in two transplantable tumours, the SCCVII/Ha tumour and the mammary carcinoma 16C. However, only 2 out of 12 (17%) spontaneous tumours showed a similar vascular steal with hydralazine (Wood et al, 1992). In another report, only 4 out of 11 (36%) primary radiation-induced murine tumours, with doubling times of 10–84 days, showed vascular steal with 5 mg kg$^{-1}$ hydralazine but after transplantation of one of the non-steal primary tumours into isogeneic mice, 16 out of 17 (94%) transplanted tumours, with a doubling time of 4–17 days, showed steal (Field et al, 1991). These authors suggest that the most likely explanation for different responses to hydralazine was an effect of transplantation, causing the vasculature developing in a transplanted tumour to be different from that in primaries. Differing tumour responses to hypoxia may also have some contribution to the $^{31}$P-MRS changes observed. Our data suggest that tumour lag time and growth rate may be important determinants of response to vasodilators, as well as transplantation per se.

With respect to tumour vasculature, Rowell et al (1990) showed that human tumour blood flow increased in response to hydralazine (0.37–2.86 mg kg$^{-1}$), suggesting that human tumours contain vessels with smooth muscle and tone, perhaps as a consequence of their slow growth rate. Our data suggests that early-transplant tumours with a slow growth rate, such as the $T40_4$ and $T237_4$ tumours used herein, are a more appropriate model of human tumour vasculature than more rapidly growing, repeatedly transplanted tumours.

### ACKNOWLEDGEMENTS

This work was supported by the Cancer Research Campaign (CRC) Programme Grant (no. 1971/0404) and ZENECA Pharmaceuticals. SPR was a CRC/CRCT/ZENECA student when these studies were performed. We thank Rick Skilton and his staff for care of the animals.

### REFERENCES

Bhujwalla ZM (1988) P-31 Magnetic Resonance Spectroscopy in Cancer Therapy: a study using transplanted animal tumour models. PhD Thesis. London University.

Bhujwalla ZM, Tozer GM, Field SB, Maxwell RJ and Griffiths JR (1990*a*) The energy metabolism of RIF-1 tumours following hydralazine. *Radiother Oncol* **19**: 281–291

Bhujwalla ZM, Tozer GM, Field SB, Proctor E, Busza A and Williams SR (1990*b*) The combined measurement of blood flow and metabolism in RIF-1 tumours *in vivo*. A study using $H_2$ flow and $^{31}$P NMR spectroscopy. *NMR Biomed* **3**: 178–183

van den Boogaart A, Howe FA, Rodrigues LM, Stubbs M, Griffiths JR (1995) *In vivo* $^{31}$P MRS: absolute concentrations, signal-to-noise and prior knowledge. *NMR Biomed* **8**: 87–93

Burney IA, Maxwell RJ, Griffiths JR and Field SB (1992) Deuterium nuclear magnetic resonance imaging of the developmental pattern of tumour blood flow. In *Angiogenesis: Key Principles – Science – Technology – Medicine*, Steiner R, Weisz PB and Langer R. (eds), pp. 357–361. Birkhauser: Basle

Denekamp J (1992) The choice of experimental models in cancer research: the key to ultimate success or failure? *NMR Biomed* **5**: 234–237

Dunn JF, Frostick S, Adams GE, Stratford IJ, Howells N, Hogan G and Radda GK (1989) Induction of tumour hypoxia by a vasoactive agent. A combined NMR and radiobiological study. *FEBS Lett* **249**: 343–347

Evanochko WT, Sakai TT, Ng TC, Krishna NR, Kim HD, Zeidler RB, Ghanta VK, Brockman RW, Schiffer LM, Braunschweiger PG and Glickson JD (1984) NMR study of *in vivo* RIF-1 tumors. Analysis of perchloric acid extracts and identification of $^{1}$H, $^{31}$P and $^{13}$C resonances. *Biochim Biophys Acta* **805**: 104–116

Evelhoch JL, Sapareto SA, Nussbaum GH and Ackerman JJH (1986) Correlations between $^{31}$P NMR spectroscopy and $^{15}$O perfusion measurements in the RIF-1 murine tumor *in vivo*. *Radiat Res* **106**: 122–131

Falk P (1982) Differences in vascular pattern between the spontaneous and the transplanted C3H mouse mammary carcinoma. *Eur J Cancer Clin Oncol* **18**: 155–165

Field SB, Needham S, Burney IA, Maxwell RJ, Coggle JE and Griffiths JR (1991) Differences in vascular response between primary and transplanted tumours. *Br J Cancer* **63**: 723–726

Griffiths JR, Cady E, Edwards RHT, McCready VR, Wilkie DR and Wiltshaw E (1983) $^{31}$P-NMR studies of a human tumour *in situ*. *Lancet* **1**: 1435–1436

Horsman MR, Christensen KL and Overgaard J (1992). Relationship between the hydralazine-induced changes in murine tumor blood supply and mouse blood pressure. *Int J Radiat Oncol Biol Phys* **22**: 455–458

Horsman MR, Nordsmark M, Hoyer M and Overgaard J (1995) Direct evidence that hydralazine can induce hypoxia in both transplanted and spontaneous murine tumours. *Br J Cancer* **72**: 1474–1478

Jain RK (1988) Determinants of tumor blood flow: a review. *Cancer Res* **48**: 2641–2658

Jain RK and Ward-Hartley K (1984) Tumor blood flow – characterization, modifications, and role in hyperthermia. *IEEE Trans Sonics Ultrason* **SU-31**: 504–526

Jirtle RL (1988) Chemical modification of tumour blood flow. *Int J Hyperthermia* **4**: 355–371

Kalmus J, Okunieff P and Vaupel P (1990) Dose-dependent effects of hydralazine on microcirculatory function and hyperthermic response of murine FSall tumors. *Cancer Res* **50**: 15–19

Larcombe-McDouall JB, Mattiello J, McCoy CL, Simpson NE, Seyedsadr M and Evelhoch JL (1991) Size dependence of regional blood flow in murine tumours using deuterium magnetic resonance imaging. *Int J Radiat Biol* **60**: 109–113

Lilly MB, Katholi CR and Ng TC (1985) Direct relationship between high-energy phosphate content and blood flow in thermally treated murine tumors. *J Natl Cancer Inst* **75**: 885–889

McCredie JA, Inch WR and Sutherland RM (1971) Differences in growth and morphology between the spontaneous C3H mammary carcinoma in the mouse and its syngeneic transplants. *Cancer* **27**: 635–642

Maxwell RJ, Workman P and Griffiths JR (1989) Demonstration of tumor-selective retention of fluorinated nitroimidazole probes by $^{19}$F magnetic resonance spectroscopy *in vivo*. *Int J Radiat Oncol Biol Phys* **16**: 925–929

Menke H and Vaupel P (1988) Effect of injectable or inhalational anesthetics and of neuroleptic, neuroleptanalgesic, and sedative agents on tumor blood flow. *Radiat Res* **114**: 64–76

Moulder JE and Rockwell S (1984) Hypoxic fractions of solid tumors: experimental techniques, methods of analysis, and a survey of existing data. *Int J Radiat Oncol Biol Phys* **10**: 695–712

Negendank W (1992) Studies of human tumors by MRS: a review. *NMR Biomed* **5**: 303–324

Ng TC, Evanochko WT, Hiramoto RN, Ghanta V, Lilly MB, Lawson AJ, Corbett TH, Durant JR and Glickson JD (1982) $^{31}$P NMR spectroscopy of *in vivo* tumors. *J Magn Reson* **49**: 271–286

Nordsmark M, Maxwell RJ, Wood PJ, Stratford IJ, Adams GE, Overgaard J, Horsman MR (1996) Effect of hydralazine in spontaneous tumours assessed by oxygen electrodes and $^{31}$P magnetic resonance spectroscopy. *Br J Cancer* **74**(suppl. XXVII): S232–S235

Okunieff PG, Koutcher JA, Gerweck L, McFarland E, Hitzig B, Urano M, Brady T, Neuringer L and Suit HD (1986) Tumor size dependent changes in a murine fibrosarcoma: use of *in vivo* $^{31}$P NMR for non-invasive evaluation of tumor metabolic status. *Int J Radiat Oncol Biol Phys* **12**: 793–799

Okunieff P, Kallinowski F, Vaupel P and Neuringer LJ (1988) Effects of hydralazine-induced vasodilation on the energy metabolism of murine tumors studied by *in vivo* $^{31}$P-nuclear magnetic resonance spectroscopy. *J Natl Cancer Inst* **80**: 745–750

Ordidge RJ, Connelly A and Lohman JAB (1986) Image-selected *in vivo* spectroscopy (ISIS). A new technique for spatially selective NMR spectroscopy. *J Magn Reson* **66**: 283–294

Prichard JW, Alger JR, Behar KL, Petroff OAC and Shulman RG (1983) Cerebral metabolic studies *in vivo* by $^{31}$P NMR. *Proc Natl Acad Sci (USA)* **80**: 2748–2751

Rofstad EK, Howell RL, Demuth P, Ceckler TL and Sutherland RM (1988*a*) $^{31}$P NMR spectroscopy *in vivo* of two murine tumor lines with widely different fractions of radiobiologically hypoxic cells. *Int J Radiat Biol* **54**: 635–649

Rofstad EK, DeMuth P and Sutherland RM (1988*b*) $^{31}$P NMR spectroscopy measurements of human ovarian carcinoma xenografts: relationship to tumour

© Cancer Research Campaign 1998 

volume, growth rate, necrotic fraction and differentiation status. *Radiother Oncol* **12**: 315–326

Rowell NP, Flower MA, McCready VR, Cronin B and Horwich A (1990) The effects of single dose oral hydralazine on blood flow through human lung tumours. *Radiother Oncol* **18**: 283–292

Sansom JM and Wood PJ (1994) $^{31}$P MRS of tumour metabolism in anaesthetized vs conscious mice. *NMR Biomed* **7**: 167–171

Steel GG (1977) Growth rate of tumours. In *Growth Kinetics of Tumours*, Steel GG (ed), pp. 5–55. Clarendon: Oxford

Thomlinson RH and Gray LH (1955) The histological structure of some human lung cancers and the possible implications for radiotherapy. *Br J Cancer* **9**: 539–549

Tozer GM, Bhujwalla ZM, Griffiths JR and Maxwell RJ (1989) Phosphorus-31 magnetic resonance spectroscopy and blood perfusion of the RIF-1 tumor following X-irradiation. *Int J Radiat Oncol Biol Phys* **16**: 155–164

Twentyman PR, Brown JM, Gray JW, Franko AJ, Scoles MA and Kallman RF (1980) A new mouse tumor model system (RIF-1) for comparison of end-point studies. *J Natl Cancer Inst* **64**: 595–604

Vaupel P, Kallinowski F and Okunieff P (1989*a*) Blood flow, oxygen and nutrient supply, and metabolic microenvironment of human tumors: a review. *Cancer Res* **49**: 6449–6465

Vaupel P, Okunieff P, Kallinowski F and Neuringer LJ (1989*b*) Correlations between $^{31}$P-NMR spectroscopy and tissue $O_2$ tension measurements in a murine fibrosarcoma. *Radiat Res* **120**: 477–493

Vaupel P, Schaefer C and Okunieff P (1994). Intracellular acidosis in murine fibrosarcomas coincides with ATP depletion, hypoxia, and high levels of lactate and total Pi. *NMR Biomed* **7**: 128–136

van der Veen JWC, de Beer R, Luyten PR and van Ormondt D (1988) Accurate quantification of *in vivo* $^{31}$P NMR signals using the variable projection method and prior knowledge. *Magn Reson Med* **6**: 92–98

Warren BA (1979) The vascular morphology of tumors. In *Tumor Blood Circulation*, Petersen HI. (ed), pp. 1–47. CRC Press: Boca Raton, FL

Wood PJ, Stratford IJ, Sansom JM, Cattanach BM, Quinney RM and Adams GE (1992) The response of spontaneous and transplantable murine tumors to vasoactive agents measured by $^{31}$P magnetic resonance spectroscopy. *Int J Radiat Oncol Biol Phys* **22**: 473–476

*© Cancer Research Campaign 1998*